# Ethical and logistical imperatives for AI-driven cardiovascular risk prediction among older adults in Tanzania: framing a digital health agenda for low-income settings

**DOI:** 10.3389/fragi.2025.1673926

**Published:** 2025-12-18

**Authors:** Innocent Arnold Tesha

**Affiliations:** Geriatrics Medicine Department, First Affiliated Hospital of Jinzhou Medical University, Jinzhou, Liaoning, China

**Keywords:** artificial intelligence, cardiovascular risk prediction, older adults, digital health, geriatric cardiology, aging, Tanzania, low- and middle-income countries

## Introduction

The global increase in life expectancy, which was initially evident in developed countries, has recently begun to manifest in developing countries. This trend is primarily driven by two key factors: declining fertility rates and increasing life expectancy (O’Sullivan, 2023). Africa is also experiencing this demographic shift, driven by increasing life expectancy and a rising burden of non-communicable diseases, particularly cardiovascular disease (CVD) among older adults (Minja et al., 2022). Similarly, Tanzania is undergoing a significant demographic shift, marked by rapidly increasing life expectancy and increasing rates of non-communicable diseases, particularly CVD in older adults (Bennett et al., 2018). This shift is attributed to several factors, including aging population: the proportion of older adults in Tanzania has increased from 4% in the 2012 to 5.7% in the 2022 census, indicating a significant increase in the aging population over that decade (Findings, 2022). Urbanization: Tanzania’s rapid urbanization has led to changes in lifestyle and living conditions such as sedentary lifestyles, unhealthy diets, and increased exposure to environmental pollutants (United Republic of Tanzania, 2014). Collectively, these factors have contributed to a surge in CVD and diabetes across both urban and rural communities (Hamid et al., 2019).

Recent national data for Tanzania reveal a severe and growing CVD epidemic. According to the 2023 Tanzania STEPS Survey, 23.1% of adults are living with hypertension. The health crisis escalates dramatically across age groups as prevalence surges to 43.5% in adults aged 46 and older, marking a nearly 3.5-fold increase in risk for older populations (Non-communicable Disease Risk Factors STEPS, 2023) Yet, fewer than 34.3% of hypertensive individuals are aware of their condition, and only one-third receive continuous treatment (Muhihi et al., 2020) These trends illustrate the urgent need for innovative risk prediction and prevention strategies.

Artificial intelligence (AI), particularly machine learning models for cardiovascular risk prediction, has gained global traction as a tool to enhance preventive care and offer transformative potential from early intervention to optimized resource allocation (Kasartzian and Tsiampalis, 2025). In high-income countries, AI-driven tools are already being integrated into electronic health systems to guide early interventions for heart failure, stroke, and coronary artery disease (Attia et al., 2019).

However, the promise of AI in resource-constrained settings like Tanzania is challenged by digital exclusion, low digital and health literacy, and limited access to continuous care. This opinion article explores logistical imperatives necessary to guide the responsible adoption of AI-driven cardiovascular risk prediction tools in Tanzania’s elderly population. We argue that without contextualized frameworks for digital inclusion, data governance, and infrastructural support, Tanzania risks forfeiting the transformative benefits of the emerging technologies. By highlighting these imperatives, we aim to contribute to ongoing discourse on digital health implementation in low-income settings.

## Materials and methods

This article adopts a descriptive approach, structured on the personal experiences of providing care to old adult patients within urban and rural communities. The analysis is structured to highlight the ongoing challenges faced by elderly patients in both urban and rural communities, emphasizing the need for ethically sound and logistically feasible AI-driven solutions for old age-associated cardiovascular diseases.

## Settings

The opinions in this article are based on both clinical and social experiences from the Dar es Salaam region. In brief, Dar es Salaam region is a relatively small but densely populated area in Tanzania located on the eastern coast of the country, covering an area of 1,393 square kilometers divided into three districts: Ilala, Kinondoni, and Temeke. According to the 2022 national census, the region’s population was 5,383,728, with a population density of approximately 3,868 people per square kilometer (Findings, 2022). This high population density is indicative of the urban nature of the region, with the majority of the population residing in urban areas.

## Results

The inception of geriatric care services in Tanzania can be traced back to the establishment of the first recognized outpatient services at Muhimbili-Mloganzila National Hospital in Kibamba, Dar es Salaam. This pioneering initiative focused on providing outpatient care services specifically designed to support older adults. However, despite this significant step forward, the facility lacked a registered geriatric specialist. This gap highlights the ongoing need for specialized geriatric expertise to ensure comprehensive and effective care for the aging population. During outpatient consultations, we observed a notable array of cardiovascular cases among the elderly population, particularly among older adults presenting with newly diagnosed chronic conditions. These diseases have become the predominant causes of hospital visits, admissions, and adverse outcomes, underscoring the significant burden of cardiovascular diseases within this demographic (Lettino et al., 2022). Among the cases encountered, one particularly noteworthy instance that underscored the necessity for early cardiovascular risk prediction involved a 73-year-old male patient, Massawe (permission granted to use real name), who presented with a history of fatigue, reduced urine output, nausea, episodes of vomiting, and mild lower limb edema. He reported no prior history of chronic illness, alcohol consumption, or cigarette smoking. Upon evaluation, he was diagnosed with stage 3 hypertension, which had progressed to chronic kidney disease and ultimately resulted in end-stage renal disease (Georgiano et al., 2021). This case highlights the critical importance of early detection and timely intervention in managing cardiovascular risks to prevent severe complications such as renal failure. During our interactions, the patient posed several insightful questions that reflect common concerns among patients facing similar conditions. He inquired about the etiology of his hypertension, seeking to understand the potential causes that led to his renal failure. He also questioned why he had remained unaware of his hypertension for so long, pondering whether there were any early signs or symptoms that he should have recognized. Finally, he asked for guidance on managing and controlling his existing hypertension to prevent further complications. These questions highlight the critical need for patient education and awareness regarding the risk factors, early signs, and management strategies for hypertension, especially in the context of preventing severe outcomes such as renal failure. In the elderly population, studies have shown that the risk of cardiovascular complications and mortality increases with blood pressure in older patients with chronic kidney disease (Yang, 2022). During routine clinic visits, we lacked an assessment tool to effectively monitor his blood pressure readings. To address this gap, we devised a simple yet practical solution by creating a small booklet to meticulously record his blood pressure readings over the subsequent 6 months. This initiative not only facilitated better tracking of his blood pressure levels but also prompted him to actively participate in his own healthcare management. In light of such cases, a critical question emerges: How many other elderly individuals, both in rural and urban settings, may be suffering from undiagnosed cardiovascular diseases such as hypertension and diabetes, which can lead to severe and unforeseen complications like end-stage renal disease, coronary heart disease, and stroke? This personal experience, along with many other comparable cases, underscores the urgent need for an affordable, innovative AI-driven and machine learning predictive model for early screening, risk assessment, and risk prediction in low-income rural and urban communities across Tanzania and Africa.

## Discussion

Cardiovascular disease is a leading cause of morbidity and mortality among the elderly, posing significant challenges to healthcare systems worldwide (Díez-Villanueva et al., 2022). Aging leads to a decline in cardiovascular function through changes such as increased arterial stiffness, left ventricular hypertrophy, and diastolic dysfunction. These changes contribute to a higher prevalence of hypertension, heart failure, and arrhythmias in the elderly population (Ciumărnean et al., 2022). The elderly population in particular is at higher risk due to age-related physiological changes and the presence of multiple comorbidities, which can complicate the management of CVD (Benjamin et al., 2019). Artificial intelligence has emerged as a transformative force in the fields of aging and cardiovascular medicine, offering promising tools to predict and manage cardiovascular risk with an accurate precision. AI-driven models are able to synthesize large, multidimensional datasets that integrate diverse data types such as clinical biomarkers, imaging, and electronic health records to uncover patterns that are often indiscernible to clinicians using conventional methods (Johnson et al., 2018; Topol, 2019). In the context of aging populations, AI has shown particular value in early detection of sub-clinical disease and forecasting adverse outcomes among older adults. Beyond traditional risk-score augmentation via machine learning algorithms such as random forests or gradient boosting whose predictive accuracy often surpasses conventional tools like the Framingham or ASCVD score when trained on diverse datasets, recent innovations are extending the frontier of care. Deep learning frameworks have emerged that estimate “vascular age” from photoplethysmography signals with demonstrated associations with cardiovascular events. Multi-modal systems that integrate data from various sources, such as wearable sensors, medical imaging, and mobile-data streams, are being deployed as digital twins of older patients, enabling continuous monitoring and dynamic risk stratification. Meanwhile, ambient motion- and behavior-tracking platforms in assisted-living settings leverage computer-vision and anomaly-detection algorithms to anticipate falls or heart-failure decompensation. Together, these advances position AI not just as a static risk calculator but as a real-time predictive ecosystem tailored to aging populations and their complex comorbidities (Weng et al., 2017; Naik et al., 2025). These models can continuously improve by learning from new inputs, enabling dynamic adaptation to changing epidemiological trends, comorbidities, care environments, and critical features for managing multi-morbidity in older adults.

**FIGURE 1:**
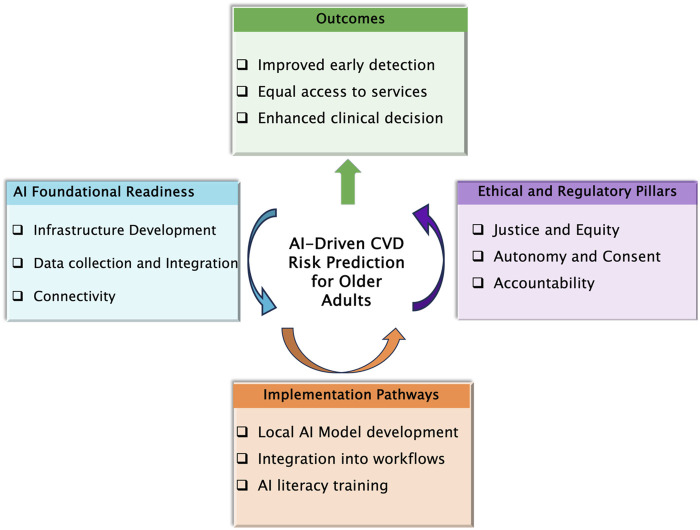
Conceptual framework illustrating the ethical and logistical pathways required for implementing AI-driven cardiovascular risk prediction in older adults within low-income settings such as Tanzania.

Although artificial intelligence offers a transformative opportunity for cardiovascular risk prediction and elderly care, its successful adoption in Tanzania’s elderly healthcare landscape is constrained by significant logistical barriers. These structural and operational challenges—such as limited digital infrastructure, particularly in rural and peri-urban settings where the majority of elderly Tanzanians reside—risk rendering AI tools ineffective and unsustainable if left unaddressed, particularly in the context of aging populations with complex care needs. Furthermore, the integration of AI into routine geriatric care is significantly impeded by fragmented and paper-based health information systems. These systems are characterized by a lack of interoperability and data sharing, which complicates the seamless integration of AI-driven solutions. Elderly patients often receive care from multiple providers, resulting in disjointed data trails that hinder the development of comprehensive longitudinal risk models. This fragmentation of care and data management poses a significant challenge to the effective implementation of AI in geriatric care. Additionally, the critical shortage of healthcare professionals proficient in AI, particularly in the fields of geriatrics, data science, and biomedical informatics, further exacerbates the challenges of adopting AI technologies. The absence of specific guidelines for the evaluation, approval, and monitoring of AI-based health technologies has hampered its wider use. These constraints collectively form major barriers to the successful implementation and adoption of AI in Tanzania’s healthcare system, particularly in the context of geriatric care. Beyond logistical constraints, the integration of AI-driven cardiovascular risk prediction among Tanzania’s older adult population presents not only a technological opportunity but also a profound ethical imperative. To ensure equitable and responsible deployment, three core ethical principles: justice, accountability, and data sovereignty must guide implementation. Ethical justice requires universal availability of AI-driven health benefits, regardless of geographic or socioeconomic status. Accountability and data governance further define the imperative of preventing data misuse, biased predictions, or algorithmic errors to safeguard patient welfare and public trust. Finally, the principle of autonomy requires that older adults demonstrate meaningful understanding and provide informed consent for the use of AI in their care. Building upon these ethical and logistical imperatives, Figure 1 presents a conceptual framework outlining a structured pathway for the implementation of AI-driven cardiovascular risk prediction in older adults in low-income settings. The case study presented underscores the potential for substantial improvements in the management of CVD among the elderly through the adoption of AI-driven and machine learning risk prediction models. These advanced technologies offer the promise of early screening, prevention, and intervention, which are crucial for mitigating the burden of CVD in this vulnerable population. By leveraging AI and machine learning, healthcare providers can enhance the early detection of CVD, facilitate timely interventions, and improve overall health outcomes for older adults. To address the challenges, several strategic measures could be implemented. One such measure involves the development of AI tools using locally generated datasets. These datasets should be sourced from a variety of healthcare settings, including outpatient clinics, primary healthcare centers, and regional, zonal and, tertiary hospitals. Utilizing such diverse and locally relevant data can significantly enhance the accuracy of AI models and mitigate the risk of algorithmic bias, ensuring that the models are well-calibrated to the specific health profiles and needs of the Tanzanian population. Furthermore, the establishment of national elderly health registries or the integration of geriatric indicators into the country’s existing digital health platforms would provide a robust foundation for AI model training, validation, and monitoring. These registries would serve as comprehensive repositories of health data specific to the elderly, facilitating the development of more targeted and effective AI-driven intervention. By incorporating geriatric indicators into digital health platforms, healthcare providers can better track and manage the health outcomes of older adults, thereby improving the overall quality of care (Rentsch et al., 2017).

### Limitations

This opinion article is grounded in personal narratives and analyses of interactions with patients requiring preventive care. Although these accounts provide valuable qualitative insights, they may not fully capture the quantitative burden of cardiovascular diseases among the elderly in both rural and urban communities. Future research should prioritize establishing the prevalence and impact of CVD in this elderly population. Such investigations could offer deeper insights into the general landscape of CVD management and the potential for technology-driven interventions in this demographic.

## Conclusion

AI-driven cardiovascular risk prediction continues to advance rapidly in high-income countries, offering new paradigms for proactive, personalized, and efficient healthcare delivery (WHO, 2021). However, in low-income countries such as Tanzania, especially within elderly care, practical implementation of such technologies remains fragmented. Although digital health strategies have been developed at the national level (United Republic of Tanzania, 2019), operationalization within primary care where most elderly patients seek services is limited by systemic barriers and the absence of AI-specific governance frameworks. A call to action is therefore made to national health authorities, policymakers, healthcare workers, and stakeholders to prioritize the development and implementation of contextually adapted and age-sensitive digital health interventions. These efforts must prioritize the elderly population in Tanzania in an inclusive and sustainable way, particularly in underserved rural communities.

Proposed framework for ethical and logistical integration of AI-driven cardiovascular risk prediction in older adults in low-income settings.

## References

[B1] Attia Z. I. Noseworthy P. A. Lopez-Jimenez F. Asirvatham S. J. Deshmukh A. J. Gersh B. J. (2019). An artificial intelligence-enabled ECG algorithm for the identification of patients with atrial fibrillation during sinus rhythm: a retrospective analysis of outcome prediction. Lancet 394 (10201), 861–867. 10.1016/S0140-6736(19)31721-0 31378392

[B2] Benjamin E. J. Muntner P. Alonso A. Bittencourt M. S. Callaway C. W. Carson A. P. (2019). Heart disease and stroke Statistics-2019 update: a report from the American heart association. Circulation 139 (10), e56–e528. 10.1161/CIR.0000000000000659 30700139

[B3] Bennett J. E. Asirvatham S. J. Deshmukh A. J. (2018). NCD countdown 2030: worldwide trends in non-communicable disease mortality and progress towards sustainable development goal target 3.4. Lancet Publishing Group. 10.1016/S0140-6736(18)31992-5 30264707

[B4] Ciumărnean L. Milaciu M. V. Negrean V. Orăşan O. H. Vesa S. C. Sălăgean O. (2022). Cardiovascular risk factors and physical activity for the prevention of cardiovascular diseases in the elderly. Int. J. Environ. Res. Public Health, 19, 207. 10.3390/ijerph19010207 PMC8751147 35010467

[B5] Díez-Villanueva P. Jiménez-Méndez C. Bonanad C. García-Blas S. Pérez-Rivera A. Allo G. (2022). Risk factors and cardiovascular disease in the elderly. IMR Press Limited. 10.31083/j.rcm2306188 PMC11273864 39077174

[B6] Findings K. (2022). The United Republic of Tanzania basic demographic and socio-economic profile key findings 2022 population and housing census.

[B7] Georgianos P. I. Agarwal R. (2021). Hypertension in chronic kidney disease (CKD): diagnosis, classification, and therapeutic targets. Oxford University Press. 10.1093/ajh/hpaa209 PMC8057136 33331853

[B8] Hamid S. Groot W. Pavlova M. (2019). Trends in cardiovascular diseases and associated risks in sub-Saharan Africa: a review of the evidence for Ghana, Nigeria, South Africa, Sudan and Tanzania. Aging Male 22 (3), 169–176. 10.1080/13685538.2019.1582621 30879380

[B9] Johnson K. W. Torres Soto J. Glicksberg B. S. Shameer K. Miotto R. Ali M. (2018). “Artificial intelligence in cardiology.” J. Am. Coll. Cardiol. 12, 2668–2679. 10.1016/j.jacc.2018.03.521 29880128

[B10] Kasartzian D. I. Tsiampalis T. (2025). Transforming cardiovascular risk prediction: a review of machine learning and artificial intelligence innovations. Life, 15, 94. 10.3390/life15010094 39860034 PMC11766472

[B11] Lettino M. Mascherbauer J. Nordaby M. Ziegler A. Collet J. P. Derumeaux G. (2022). “Cardiovascular disease in the elderly,” in Proceedings of the european society of cardiology - Cardiovascular round table. Oxford University Press. 10.1093/eurjpc/zwac033 35167666

[B12] Minja N. W. Nakagaayi D. Aliku T. Zhang W. Ssinabulya I. Nabaale J. (2022). Cardiovascular diseases in Africa in the twenty-first century: gaps and priorities going forward. Front. Cardiovasc. Med. 10 (9), 1008335. 10.3389/fcvm.2022.1008335 PMC9686438 36440012

[B13] Muhihi A. J. Anaeli A. Mpembeni R. N. M. Sunguya B. F. Leyna G. Kakoko D. (2020). Prevalence, awareness, treatment, and control of hypertension among young and middle-aged adults: results from a community-based survey in rural Tanzania. Int. J. Hypertens. 2020, 9032476. 10.1155/2020/9032476 32963821 PMC7491449

[B14] Naik A. Nalepa J. Wijata A. M. Mahon J. Mistry D. Knowles A. T. (2025). Artificial intelligence and digital twins for the personalised prediction of hypertension risk. Comput. Biol. Med. 196 (Sep), 110718. 10.1016/j.compbiomed.2025.110718 40628170

[B15] Non-communicable Disease Risk Factors (STEPS) (2023). Survey 2023 country report.

[B16] O’Sullivan J. N. (2023). Demographic delusions: world population growth is exceeding Most projections and jeopardising scenarios for sustainable futures. World 4 (3), 545–568. 10.3390/world4030034

[B17] Rentsch C. T. Reniers G. Kabudula C. Machemba R. Mtenga B. Harron K. (2017). Point-of-contact interactive record linkage (PIRL) between demographic surveillance and health facility data in rural Tanzania. Int. J. Popul. Data Sci. 2 (1), 3. 10.23889/ijpds.v2i1.408 30613799 PMC6314455

[B18] Topol E. J. (2019). High-performance medicine: the convergence of human and artificial intelligence. Nat. Med. 25, 44–56. 10.1038/s41591-018-0300-7 30617339

[B19] United Republic of Tanzania (2014). The United Republic of Tanzania basic demographic and socio-economic profile key findings 2014 2012 population and housing census.

[B20] United Republic of Tanzania (2019). The United Republic of Tanzania digital health strategy.

[B21] Weng S. F. Reps J. Kai J. Garibaldi J. M. Qureshi N. (2017). Can machine-learning improve cardiovascular risk prediction using routine clinical data? PLoS One 12 (4), e0174944. 10.1371/journal.pone.0174944 28376093 PMC5380334

[B22] WHO (2021). Ethics and governance of artificial intelligence for health: WHO guidance. World Health Organization.

[B23] Yang J. W. (2022). Blood pressure control in elderly chronic kidney disease patients. Electrolyte Blood Press. 20 (2), 57–63. 10.5049/EBP.2022.20.2.57 36688210 PMC9827045

